# Effect of the feeding system on the fatty acid composition, expression of the Δ^9^-desaturase, Peroxisome Proliferator-Activated Receptor Alpha, Gamma, and Sterol Regulatory Element Binding Protein 1 genes in the semitendinous muscle of light lambs of the Rasa Aragonesa breed

**DOI:** 10.1186/1746-6148-6-40

**Published:** 2010-07-22

**Authors:** Elda Dervishi, Carmen Serrano, Margalida Joy, Malena Serrano, Clementina Rodellar, Jorge H Calvo

**Affiliations:** 1 Unidad de Tecnología en Producción Animal, CITA, Zaragoza, Spain; 2 Laboratorio de Génetica Bioquímica (LAGENBIO), Dpto. Anatomia, Embriología y Genética Animal, Facultad de Veterinaria (Universidad de Zaragoza), Spain; 3 Departamento de Mejora Genética Animal, INIA-Madrid, Spain; 4 Fundación ARAID

## Abstract

**Background:**

Conjugated linoleic acids (CLAs) are receiving increasing attention because of their beneficial effects on human health, with milk and meat products derived from ruminants as important sources of CLA in the human diet. *SCD *gene is responsible for some of the variation in CLA concentration in adipose tissues, and *PPARγ*, *PPARα *and *SREBP1 *genes are regulator of *SCD *gene. The aim of this work was to evaluate the effect of the feeding system on fatty acid composition, CLA content and relative gene expression of Δ^9^-desaturase (*SCD*), Peroxisome Proliferator-Activated Receptor Gamma (*PPARγ*), Peroxisome Proliferator-Activated Receptor Alpha, *(PPARα) *and Sterol Regulatory Element Binding Protein *(SREBP1) *in Rasa Aragonesa light lambs in semitendinous muscle. Forty-four single-born male lambs were used to evaluate the effect of the feeding system, varying on an intensity gradient according to the use of concentrates: 1. grazing alfalfa, 2. grazing alfalfa with a supplement for lambs, 3. indoor lambs with grazing ewes and 4. drylot.

**Results:**

Both grazing systems resulted in a higher concentration of vaccenic acid (VA), CLA, CLA/VA acid ratio, and a lower oleic content, oleic acid (C18:1)/stearic acid (C18:0) ratio, PUFA n-6/n-3 ratio and *SCD *expression compared to other diets. In addition feeding system affected the fatty acid composition and *SCD *expression, possibly due to CLA concentration or the PUFA n-6/n-3 ratio. Both expression of the *SCD *gene and the feeding system were important factors affecting CLA concentration in the animal's semitendinous muscle. *PPARγ, PPARα *and *SREBP1 *expression seemed to be unaffected by the feeding system. Although no significant results were found, *PPARγ, PPARα *and *SREBP1 *showed similar expression pattern as *SCD*. Moreover, the correlation results between *SCD *expression and *PPARγ *(p < 0.01), as well as *SREBP1 *(p < 0.01) expression, may suggest that these genes were affecting *SCD *expression in a different way.

**Conclusions:**

The data indicated that the feeding system is the main factor affecting the fatty acid composition and *SCD *gene expression, which is also affected by CLA and possibly by n-6/n-3 PUFAs.

## Background

Conjugated linoleic acids (CLAs) are a group of positional and geometric isomers of octadecadienoic acids with conjugated double bonds. These groups of fatty acids are receiving increasing attention because of their possible beneficial effects on human health; they reduce the incidence of atherosclerosis, diabetes and cancer in animals [1-8]. Milk and meat products derived from ruminants are important sources of CLA in the human diet [9]. The biohydrogenation of polyunsaturated fatty acids (PUFAs) that takes place in the rumen by microbial activity leads to an increase of saturated fatty acids (SFA), as well as their intermediate products. Some of the PUFAs and these intermediate products escape biohydrogenation and are incorporated into milk and body fat [10]. CLA is one of the most important intermediate products. The major isomer of CLA is *cis-9, trans-11*, which represents 80-90% of the total CLA, followed by *trans-10, cis-12*. Studies have found that the wide range of CLA's activity results from an interaction between the two major CLA isomers [7]. Ruminant CLA comes from two sources [11]: one from biohydrogenation in the rumen, and the other is derived from the synthesis from *trans-11 *C18:1 by the activity of Δ^9^-desaturase (SCD) in animal tissues [12]. The SCD protein is encoded in the ovine species by a gene located on chromosome 22 (OAR22) [13], in a region where a positional quantitative trait locus (QTL) has been detected for the CLA: C18:1 n-7 vaccenic acid ratio in milk [14]. In bovine, several studies have shown significant associations between this gene and the fatty acid composition of meat and milk [15,16]. Expression of the *SCD *gene is regulated by dietary, especially PUFA n-6 and n-3 families, hormonal and environmental factors [17] through the sterol regulatory element binding protein (SREBP) [18] and peroxisome proliferator-activated receptor proteins (PPAR) [19]. PUFAs repress human and mouse *SCD *expression, as well as other lipogenic genes, by reducing gene expression and the maturation of SREBP1 [17]. SREBPs have been established as lipid synthetic transcription factors for cholesterol and fatty acid synthesis [20], activating genes required for the synthesis and fatty acid incorporation into triacylglycerols and phospholipids, including *SCD *gene [18]. *SCD *expression was increased in livers of transgenic mice that overexpress transcriptionally active nuclear forms of human *SREBP-1a *[21]. PPARs belong to a superfamily of hormone receptors that regulate the transcription of genes involved in different lipid metabolism pathways. Three PPAR isotypes have been identified: α, β, and γ. PPARα operates in the catabolism of the fatty acids in the liver while PPARγ influence the storage of the fatty acids in the adipose tissue. PPARγ is most abundant in adipose tissue and stimulates adipocyte differentiation and lipogenesis of mature adipocytes [19]. The PPARs are activated by a number of compounds, including polyunsaturated fatty acids [22]. Dietary n-6 and n-3 PUFAs repress *SCD *and *SREBP *gene expression while PUFAs are known to activate nuclear transcription factors such as PPARs, which modulate gene expression in response to environmental and dietary factors [23]. It has been shown that activation of PPARα induces transcription of *SCD *in mice [24]. Furthermore, studies in *PPARα-*null mice showed that *PPARα *deficiency affects the response of *SCD *and *SREBP1 *mRNAs to re-feeding following starvation [25]. In the same way, *SCD *expression was highly correlated with the expression of a number of other genes that are responsive to peroxisomal proliferator-activated receptor γ (PPARγ) agonists and involved in different components of adipogenesis. Small-interference RNA-mediated *knock*-*down *of *SCD1 *in adipocytes impaired adipogenesis and decreased PPARγ protein levels [26,27]. In bovine, studies have reported that the expression of *SCD *gene is regulated by the transcription factors SREBP1 [28,29], PPARα [29] and PPARγ [28,29].

In sheep, only a few studies have investigated the nutritional regulation of *SCD *gene expression, finding that the increased concentration of CLA in lambs fed forage-based diets was associated with both an increase in the substrate for conversion to CLA and a decrease in *SCD *gene expression as detected by northern blot [30]. Vasta et al. (2009) [31] showed that the concentrate feeding system increased the amount of saturated fatty acids (SFA), monounsaturated fatty acids (MUFA) and n-6 PUFA as well as decreased the amount of n-3 PUFA when compared to forage-fed animals but did not affect SCD protein expression. Therefore, the objective of this study was to evaluate the effect of the feeding system on fatty acid composition, CLA content and *SCD, PPARγ, PPARα *and *SREBP1 *gene expression and their relationship in semitendinous muscle in light lambs.

## Results and discussion

### Fatty acid composition

**Figure 1:**
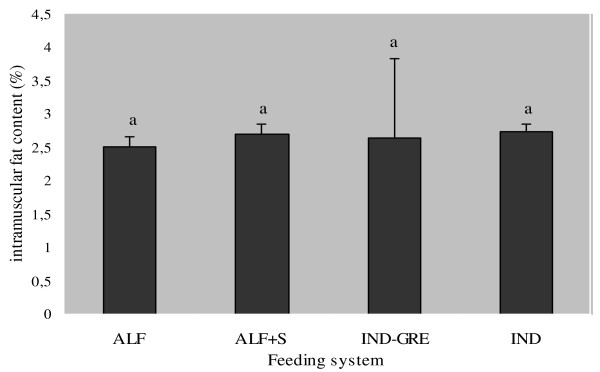
**Total intramuscular fat content (%) according to feeding system**. ALF: Grazing Alfalfa; ALF+S: Grazing alfalfa with supplement for lambs; IND-GRE: Indoor lambs with grazing ewes; and IND: Indoors. Different subscripts differ with at least *P < 0.05*.

**Figure 2:**
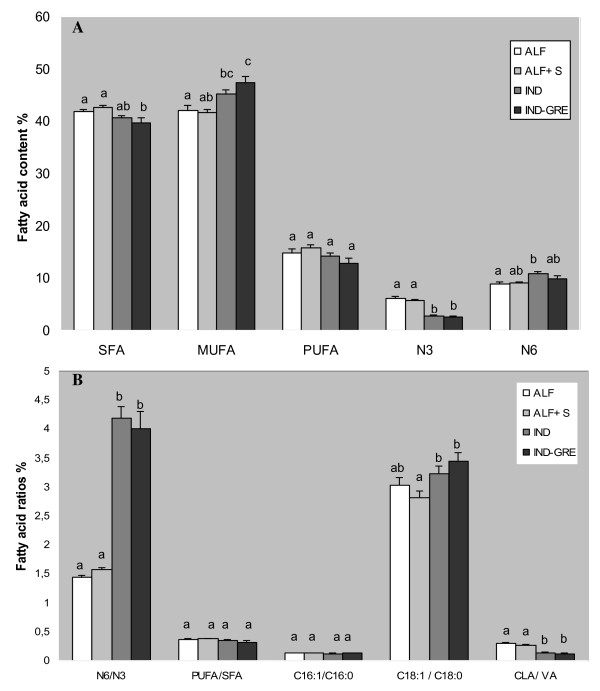
**Mean fatty acid composition of the semitendinous muscle in Rasa Aragonesa lambs for each feeding system**. A: Mean values of total satured (SFA), monounsatured (MUFA) and polyunsatured (PUFA) fatty acids, and n-3 and n-6 PUFA composition (expressed as the percentage of total fatty acids). B: Fatty acid ratios. ALF: Grazing Alfalfa; ALF+S: Grazing alfalfa with supplement for lambs; IND-GRE: Indoor lambs with grazing ewes; and IND: Indoors. Different subscripts differ with at least P < 0.05. The ratio of PUFA/SFA was calculated as (C18:2+C18:3+C20:4+C20:5+C22:4+C22:5+C22:6)/(C10:0+ C12:0+C14:0+C16:0+C18:0+ C20:0). The ratio of n-6/n-3 was calculated as (C18:2 n-6+C20:4 n-6+C22:4 n-6)/(C18:3 n-3+ C20:5 n-3+ C22:5 n-3+ C22:6 n-3.

The results of total amount of intramuscular fat showed no significant differences among diets (Figure 1). Intramuscular fatty acid composition of semitendinous muscle for each feeding system is reported in Table 1 and Figure 2.

**Table 1:** Mean fatty acid composition (expressed as the percentage of total fatty acids) of the semitendinous muscle in Rasa Aragonesa lambs for each feeding system^1-2^.

Fatty acid	Feeding system				*SE*
	ALF	ALF+S	IND-GRE	IND	
C10:0	0.26^bc^	0.27^c^	0.21^ab^	0.17^a^	0.020
C12:0	0.62^a^	0.63^a^	0.39^b^	0.28^b^	0.057
C14:0	5.72^a^	5.66^a^	4.03^b^	3.31^b^	0.349
C16:0	22.61^a^	22.66^a^	22.87^a^	22.54^a^	0.381
C16:1	3.04^a^	2.88^a^	2.81^a^	2.86^a^	0.113
C17:0	1.18^a^	1.18^a^	1.50^b^	1.701^b^	0.093
C17:1	0.89^ab^	0.84^a^	1.10^bc^	1.28^c^	0.080
C18:0	11.38^a^	12.06^a^	11.63^a^	11.678^a^	0.507
C18:1 n-9	34.12^ab^	33.57^a^	37.23^bc^	39.70^c^	1.203
C18:1 n-7	3.94^ab^	4.18^b^	3.93^ab^	3.48^a^	0.188
C18:2 n-6	6.43^a^	6.89^ab^	8.22^b^	7.35^ab^	0.526
C18:3 n-3	2.56^a^	2.58^a^	0.82^b^	0.65^b^	0.139
C18:2 cis9-trans11	1.17^a^	1.14^a^	0.55^b^	0.43^b^	0.074
C 20:0	0.09^a^	0.09^a^	0.07^b^	0.07^b^	0.005
C 20:1 n-9	0.09^a^	0.10^a^	0.10^a^	0.10^a^	0.006
C 20:4 n-6	2.28^a^	2.04^a^	2.39^a^	2.35^a^	0.208
C 20: 5 n-3	1.30^a^	1.16^a^	0.59^b^	0.54^b^	0.109
C22:4 n-6	0.08^a^	0.08^a^	0.17^b^	0.17^b^	0.013
C22:5 n-3	1.38^a^	1.26^a^	0.85^b^	0.81^b^	0.108
C22:6 n-3	0.86^a^	0.73^ab^	0.54^b^	0.52^b^	0.094

^1^Different subscripts differ with at least P < 0.05. SE is standard error

^2 ^ALF: Grazing Alfalfa; ALF+S: Grazing alfalfa with supplement for lambs; IND-GRE: Indoor lambs with grazing ewes; and IND: Indoors.

### Saturated fatty acids (SFAs)

The feeding system had a significant effect on most of the SFAs (P < 0.05) except for the major ones, palmitic and estearic acids. Both grazing treatments, ALF and ALF+S, presented greater C12:0, C14:0 and C20:0 levels than did the IND and IND-GRE treatments. There is some controversy about the effect of the inclusion of a concentrate in the diet on the SFA content. Several studies have found that SFA increased with concentrate intake [32,33], whereas others did not find this effect [31,34,35]. Feeding systems include two main concepts: diet and rearing management. In grazing treatments lambs kept suckling and grazing until slaughter. In these lambs, the SFA content of muscle could be influenced by milk composition instead of fresh forage from grazing.

The total SFA content was lower in IND treatment and was significantly different from the ALF and ALF+S treatments (P < 0.05) (Figure 2A). An increase in the SFA content was expected when the proportion of concentrate in the diet was increased [36]. Factors like breed, slaughter weight and type of muscle can influence muscle CLA concentrations [37]. In the present study, animals were slaughtered at 22-24 kg, younger than 90 days old, and the muscle used to estimate the fatty acid composition was semitendinous instead of the longissimus dorsi. Muscles differ in fat concentration because of their function and their location within the body, which emphasizes the importance of investigating more than one muscle [38].

### Monounsaturated fatty acids (MUFAs)

The type of feeding system used affected the C17:1, C18:1 n-9, and C18:1 n-7 concentration (P < 0.05), whereas the C16:1 and C20:1 n-9 contents were not affected (P > 0.05). Oleic acid (C 18:1 n-9) content was higher in both indoor systems, although differences were only significant between the alfalfa grazing treatments and IND (P < 0.05; Table 1). Several studies have concluded that grazing lambs have a lower concentration of C16:1 and C18:1 than concentrate-fed lambs [39,40]. The latter lambs had more fat when compared to those that grazed, which was usually combined with an increase in oleic acid [33] mainly due to an increase in the activity of the enzyme SCD, which is also responsible for the synthesis of oleic acid C18:1 *cis-9 *from stearic acid C18:0.

Trans-vaccenic acid (C18:1 n-7) was significantly higher in the ALF+S group than in the IND group (P < 0.05). This fatty acid was significantly higher in the intramuscular fat from pasture-fed lambs [33]. Total MUFA was higher in IND and IND-GRE group (Figure 2A).

### Polyunsaturated fatty acids (PUFAs)

Linoleic acid (C18:3 n-3) content was higher in both grazing groups (ALF and ALF+S) than in indoor treatments (IND and IND-GRE; P < 0.05). Linoleic acid (C18:2 n-6) content was greater in the IND-GRE group than in the ALF group (P < 0.05). In the present study, the inclusion of concentrate in the ALF+S group did not decrease CLA concentration. The animals of ALF+S group were suckling until slaughter and this could have offset the effect of concentrate intake. The isomer *cis-9, trans-11 *of CLA was at higher levels in both grazing groups, ALF and ALF+S (P < 0.001). The differences in CLA content observed between the two alfalfa treatments and the indoor groups (IND and IND-GRE) agree with the premise that pasture grazing increases the CLA concentration [31,41].

In the present study, total PUFA contents were similar between treatments (Figure 2A). Other studies have suggested that the intramuscular fat from grazing lambs has shown a higher proportion of PUFA [33,42]. Despite the lack of differences in the total PUFA levels between treatments (P > 0.05), n-6 PUFA concentration was lower in the ALF treatment, being only significantly different from the IND-GRE treatment (P < 0.05) (Figure 2A). By contrast, the series of n-3 PUFA was significantly higher in both grazing groups, ALF and ALF+ S, when compared with the IND and IND-GRE groups (P < 0.05). This is because fresh forage has a higher concentration of n-3 PUFA than concentrate [43]. The n-6/n-3 ratio was lower in ALF and ALF+S lambs (P < 0.001) (Figure 2B). According to medical recommendations, the ratio PUFA n-6/n-3 should be lower than four in human diets. An increased consumption of n-3 PUFA has been recommended to overcome the perceived imbalance in the ratio of PUFA n-6/n-3 PUFA in human diets [43]. The values of PUFA n-6/n-3 in the animals on ALF and ALF+S diets fulfilled these recommendations, while the IND and IND-GRE groups were close to it.

No differences were found between the four groups for the ratio of PUFA/SFA, but there was a tendency for the ratio to be higher in ALF and ALF+S groups (P = 0.119) (Figure 2B).

We calculated three desaturation indexes [11]: palmitoleic acid (C16:1)/palmitic acid (C16:0), oleic acid (C18:1)/stearic acid (C18:0) and CLA/VA acid (Figure 2B). For the ratio of palmitoleic acid C16:1/palmitic acid C16:0, no statistically significant differences (P > 0.05) were found between groups. The ratio of C18:1/stearic acid C18:0 was higher in the IND group than in the ALF+S group (P < 0.05), while the ratio of CLA/VA acid was higher in the ALF group and ALF+S group when compared to those of the IND and IND-GRE groups (P < 0.01).

### Real-time RT-PCR and the effect of the feeding system on *SCD*, *SREBP1*, *PPARα *and *PPARγ *relative gene expression

**Figure 3:**
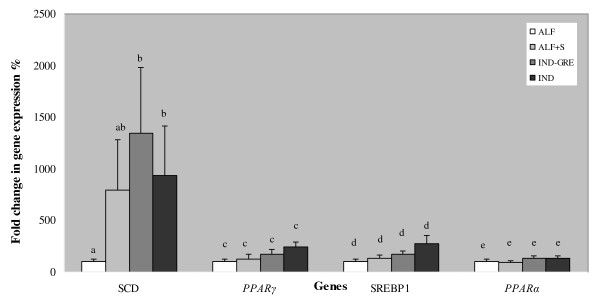
**Effect of feeding system on mRNA expression of *SCD*, *PPARγ, SREBF1 *and *PPARα *genes of Rasa Aragonesa lambs**. ALF: Grazing Alfalfa; ALF+S: Grazing alfalfa with supplement for lambs; IND-GRE: Indoor lambs with grazing ewes; and IND: Indoors. Different subscripts differ with at least *P < 0.05*.

A partial ovine genomic DNA sequence of 425 bp for *SREBP1 *was obtained (GenBank Acc. No. GU206528). The sequences of sixth and seventh exon in sheep were deduced based on the sequence of the bovine *SREBP1 *gene. Primers for real-time RT-PCR were designed from specific ovine *SREBP1 *gene. The results of the real-time RT-PCR analysis showed that *SCD *expression was significantly modulated by the feeding system. Lambs belonging to the ALF and ALF+S groups showed lower levels of *SCD *expression in comparison with the IND-GRE and IND lambs. Significant differences were found only between the ALF group and the IND-GRE and IND groups (P < 0.05) (Figure 3). The relative expression of *SCD *was 8-fold higher in ALF+S animals, 13-fold higher in IND-GRE animals and 8.4-fold higher in IND animals compared to the ALF animals, which were used as a control group. The same trend was found for the *PPARγ *and *SREBP1 *genes. The expression pattern of the *PPARγ *and *SREBP1*genes was similar to *SCD *gene expression, with the highest levels of expression in the IND group. No significant differences were found for *SREBP1*, *PPARγ *or *PPARα *gene expression between groups. The expression of *SREBP1 *was 1.33-fold higher in ALF+S animals, 1.73-fold higher in IND-GRE animals and 2.78-fold higher in IND animals than in ALF animals (Figure 3). The expression of *PPARG *was 1.21-fold higher in ALF+S animals, 4.17-fold higher in IND-GRE animals and 2.21-fold higher in IND animals. The expression of *PPARα *was 0.94-fold lower in ALF+S animals, 1.34-fold higher in IND-GRE animals and 1.29-fold higher in IND animals. Our results are in concordance with the results of Waters et al. [29]. They found that *SCD *mRNA expression tends to be reduced when increasing n-3 PUFA enriched fish oil supplement, but not in *PPARα *expression. While we did not find changes in *SREBP1 *mRNA expression Waters et al. found that *SREBP1 *mRNA expression decreased when animals were fed with 6% soybean oil plus 2% fish oil. This may be as results of the differences between diets used in our study and the previous one.

The composition of fatty acids stored in the fat depots reflects the action of the SCD protein on substrates like stearic acid and palmitic acid [44]. The animals of the IND and IND-GRE groups showed higher percentages of MUFA content and higher levels of *SCD *gene expression. These results are in concordance with those found by Daniel *et al*. [30], which suggested that the elevated oleic acid content of ruminant tissues in response to concentrate-rich diets is at least in part result of increased SCD activity. Furthermore, in our work, the diets that produced the highest levels of CLA (ALF and ALF+S) also suppressed *SCD *gene expression. It is hypothesized that mammary *SCD *gene expression may be regulated by the different *cis-9 *C18:1/C18:0 ratios brought to the mammary gland by the diet [45]. These ratios could be representing an approximation for SCD activity in semitendinous muscle. In the present study, *SCD *gene expression seems to be regulated by the different ratios incorporated into the muscle by the diet, where the animals of the ALF and ALF+S groups showed a higher content of VA, CLA, CLA/VA acid ratios, and lower oleic content, oleic acid (C18:1)/stearic acid (C18:0) ratios, PUFA n-6/n-3 ratios and *SCD *expression. In contrast, the animals of the IND and IND-GRE groups showed lower VA content, levels of CLA, and CLA/VA ratios, and higher oleic acid content, (C18:1)/stearic acid (C18:0) ratios, PUFA n-6/n-3 ratios and *SCD *expression levels. Correlation results are shown in Table 2. A strong negative correlation was found between oleic acid (C18:1 n- 9) and VA content, as well as CLA content, while a positive correlation was observed between oleic acid content and *SCD *gene expression as well as PUFA n- 6/n-3. As was expected, VA content showed a strong positive correlation with CLA content. On the other hand, CLA also showed a negative correlation with both *SCD *gene expression and PUFA n-6/n-3. Meanwhile, PUFA n-6/n-3 showed a positive correlation with *SCD *gene expression. Based on the correlations that exist between these variables, we propose the existence of a certain level of competition for the same metabolic pathway, which is strongly dependent on the feeding system and on C18:1 VA acid production. Therefore, under certain circumstances, or with different feeding systems, both stearic acid and VA acid may be competing for the SCD enzyme in semitendinous muscle, with C18:0 always being the most preferred substrate. Higher levels of CLA *cis-9, trans-11 *were yielded when the presence of C18: 1 *trans*-vaccenic acid was higher. *SCD *gene expression was lower in the presence of high levels of CLA and low levels of PUFA n-6/n-3. An effect of protected CLA on fatty acid composition in some tissues has been reported [46], and this was consistent with the inhibition of SCD. It may be that the accumulation of CLA *cis-9, trans-11 *and lower PUFA n-6/n-3 ratios in semitendinous muscle contribute to the relatively low expression of *SCD*. PUFAs are known to activate nuclear transcription factors such as PPARs [22,23]. PUFAs and peroxisome proliferators exert opposite effects on *SCD1 *mRNA levels in mouse liver, with PPARs acting as activators of *SCD *gene expression [24]. In the present work, expression results for *PPARγ, PPARα *and *SREBP1 *genes were not significant. However we must point out that their expression pattern was similar to *SCD *gene expression profile. No correlation between *PPARα *with *SCD *gene expression was founded. *PPARα *only showed a positive correlation with *PPARγ*. A moderate correlation was found between the expression of *SCD *and the expression of both *PPARγ *and *SREBP1*, but not between *PPARγ *and *SREBP1*. As has been shown in previous reports, expression of the *SCD *gene is downregulated by PUFAs (especially the n-6 and n-3 families), CLA, cholesterol, vitamin A, hormonal changes and peroxisomal proliferators [10,23,47-50]. Furthermore, unsaturated fatty acids are important because they play a role in cellular activity, metabolism and nuclear events that govern gene transcription [48]. In this sense, the dietary fats n-6 and n-3 PUFA, and especially arachidonic acid (C20:4 n- 6), have been shown to repress *SCD *gene expression [23,51]. If this were true, then the animals of the IND and IND-GRE groups, which showed the highest levels of, arachidonic acid (C20:4 n- 6), should have lower levels of *SCD *expression; however, the animals of the IND and IND-GRE groups showed higher levels of *SCD *expression. In most cases these studies have been carried out in mouse, where lipid metabolism is different from that in ruminants. In addition, these studies takes into account only the effect of CLA, without having a global view of the possible effects of other fatty acids or their ratios on *SCD *gene expression. In the present study the negative correlation between *SREBP1 *gene expression with CLA content and positive correlation with PUFA n- 6/n- 3 suggested that PUFA n- 6/n- 3 ratio was an important factor which may be affecting *SREBP1 *gene expression.

**Table 2:** Correlation results between the fatty acid composition and genes expression.

	C18:0	C18:1 n- 9	VA	CLA	n- 6/n-3	SCD	PPARG	PPARα	SREBP1	PPARG/SREBP1	CLA/PUFA	PUFA/SFA
18:0 ***P***		-0.135 0.382	0.206 0.179	-0.188 0.222	0.05 0.749	0.081 0.606	0.204 0.207	0.133 0.397	0.197 0.206	-0.012 0.93	-0.13 0.401	-0.165 0.284
*C18:1 n- 9* *P*			-0. 531** 0.000	-0.591** 0.000	0.708** 0.000	0.345* 0.024	0,087 0.592	0.122 0.436	0.29 0.059	-0.214 0.168	-0.356* 0.018	-0.773** 0.000
VA *P*				0.479** 0.001	-0.171 2.267	-0.096 0.54	-0.031 0.85	-0.063 0.690	-0.006 0.969	-0.041 0.768	0.363* 0.016	0.244 -0.111
CLA *P*					-0.777** 0.000	-0.387** 0.01	-0.234 0.147	-0.152 0.331	-0.304* 0.048	0.046 0.768	0.93** 0.000	0.388** 0.009
n- 6/n-3 *P*						0.405** 0.007	0.192 0.237	0.230 0.138	0.345* 0.023	-0.151 0.335	-0.678** 0.000	-0.558* 0.000
SCD *P*							0.554** 0.000	0.036 0.818	0.597** 0.000	0.021 0.893	-2.293 0.056	-0.356* 0.019
PPARG *P*								0.272* 0.013	0.154 0.344	0.673** 0.000	-0.198 0.221	-0.242 0.132
PPARα P									0.158 0.135	0.048 0.653	-0.101 0.341	-0.132 0.220
SREBP1 *P*										-0.58** 0.000	-0.191 0.22	-0.287 0.062
PPARG/SREBP1 *P*											-0.018 0.909	0.079 0.614
CLA/PUFA *P*												0.07 0.652
PUFA/SFA *P*												.

*P < 0.05, **P < 0.01

The results of GLM analysis showed that the feeding system (P < 0.01) and *SCD *expression (P < 0.05) both had a significant effect on CLA content. These results suggested that differences in *SCD *gene expression due to the feeding system contributed to CLA content differences detected in the intramuscular fat of the semitendinous muscle in animals of the Rasa Aragonesa breed. On the other hand, the results of analysis of variance for *SCD *expression showed that *PPARγ *and *SREBP1 *expression levels contribute in 26.4 and 5.6%, respectively.

Furthermore, the correlation results between CLA with both *SCD *gene expression and the PUFA n-6/n-3 ratio and the PUFA n-6/n-3 ratio with *SCD *gene expression suggested that CLA and the PUFA n-6/n-3 ratio both had an important effect on *SCD *gene expression. Thus, lambs from grazing groups showed higher levels of CLA and lower PUFA n-6/n-3 ratio, inhibiting *SCD *expression, and lambs fed concentrate-based diet showed lower CLA concentration and a higher PUFA n-6/n-3 ratio, which increased the expression of the *SCD *gene. No significant correlation between CLA/PUFA and expression of *SREBP1*/*PPARγ *was found. It seemed that there was a negative correlation between CLA/PUFA and *SCD *gene expression which may suggest that higher CLA/PUFA ratio lower was *SCD *expression. The results of positive correlation between *SCD *and *SREBP1*mRNA expression and PUFA n-6/n-3 showed that when these ratios raised in favour of n-6 PUFA the *SCD *and *SREBP1 *mRNA expression raised too. This suggested that n-6 PUFA concentrations in sheep may be increasing *SCD *mRNA expression through a mechanism which involved increasing *SREBP1 *mRNA expression. This mechanism did not involve CLA *cis-9, trans-11*. While it seemed that *PPARγ *was affecting *SCD *mRNA expression through another mechanism which did involve neither PUFA n-6/n-3 ratio nor CLA.

It may be possible the existence of a posttranscriptional regulator of *SCD *gene. However, no effect on SCD protein expression was found in lambs raised under a forage-based diet when compared to those raised under a concentrate-based diet [31]. A mechanism explaining the effect of CLA on *SCD *expression could involve a decrease of *SCD *mRNA and/or gene transcription has been proposed [52]. In hamster, the 30 amino acid N-terminal segment of the SCD protein is a motif associated with the rapid degradation of the protein [53]. Furthermore, the full-length 5' and 3' UTR sequence in bovines has been determined, and several motif sequences were found in the 3'UTR that affect mRNA stability, such as ATTTA motifs and the poly(A) signal [54]. Determining whether CLA, and specifically the *cis-9, trans-11 *isomer, decrease the expression of the *SCD *gene by reducing mRNA stability requires further investigation.

## Conclusions

Grazing lambs presented a higher content of 18:1 *trans-11 *and CLA, and a greater ratio of CLA/*trans*-vaccenic acid than did indoor lambs. Moreover, grazing animals showed lower n- 6/n- 3 ratios, which is favorable in regard to current human dietary guidelines.

The data indicated that the feeding system is the main factor affecting the fatty acid composition and *SCD *gene expression, which is also affected by CLA and possibly by n-6/n-3 PUFAs.

*PPARγ, PPARα *and *SREBP1 *gene expression seem to be unaffected by the feeding system, but the high correlation that exists between *SCD, PPARγ *and *SREBP1 *genes suggests that the feeding system may play an important role. More studies will be necessary to elucidate the effects of the feeding system on *PPARγ *and *SREBP1 *expression as well as determine the mechanism by which they alter *SCD *gene expression in semitendinious muscle.

These data indicate that the intramuscular fatty acid composition of lamb meat can be improved, from a human health perspective, by taking into account the interaction between nutrients and genes.

## Methods

### Animals and diets

The Rasa Aragonesa breed is the most common in the geografical area where the study was carried out. Forty-four Rasa Aragonesa spring single-born male lambs and their ewes were randomly allocated to four treatment groups (n = 11), taking into account the lambing date and the lamb's birthweight. The treatments were:

*1. Grazing alfalfa *(**ALF**): Lambs and ewes were continuously stocked on an alfalfa paddock. No concentrate was available to dams or lambs. Lambs suckled their mothers and grazed alfalfa (2.31 Mcal metabolizable energy; 223 g crude protein; 403 g neutral detergent fiber; 328 g acid detergent fiber, on a dry matter basis) until slaughter.

*2. Grazing alfalfa with supplement for lambs *(**ALF+S**): The same management as in ALF, but these lambs received concentrate *ad libitum *in creep feeders (4.27 Mcal metabolizable energy; 204 g crude protein; 206 g neutral detergent fiber and 38 g acid detergent fiber, on a dry matter basis) until slaughter.

*3. Indoor lambs with grazing ewes *(**IND**-**GRE**): Lambs remained indoors and ewes grazed eight hours a day (08:00 to 16:00 h). Afterwards, the ewes returned indoors where they had free access to dry unifeed (1.88 Mcal metabolizable energy; 69 g crude protein; 691 g neutral detergent fiber and 377 acid detergent fiber, on dry matter basis). Lambs were weaned at 45 days old and had free access to concentrate (4.27 Mcal metabolizable energy; 204 g crude protein; 206 g neutral detergent fiber and 38 g acid detergent fiber, on a dry matter basis).

*4. Indoors *(IND): Lambs and ewes were always kept in confinement. Ewes had free access to dry unifeed (1.88 Mcal metabolizable energy; 69 g crude protein; 691 g neutral detergent fiber and 377 acid detergent fiber, on a dry matter basis) and lambs had concentrate *ad libitum*. Lambs were managed equal to the IND-GRE treatment.

When the lambs reached 22-24 kg of live-weight (LW), they were slaughtered according to EU laws. Procedures were conducted according to the guidelines of the Council Directive 86/609/EEC (European Communities, 1986) on the protection of animals used for experimental and other scientific purposes. Light lamb production (18-24 kg live weight, younger than 90 days) represents the largest share of the lamb market in many Mediterranean countries [55]. Consumers show a greater preference for this type of meat, characterised by its pale pink colour and white fat.

Just after slaughter a piece of semitendinous muscle, which is also a valued meat by Mediterranean consumers, was cut and frozen in liquid nitrogen until RNA isolation. Carcasses were chilled at 4°C for 24 h and then semitendinous muscle was removed from the left half of the carcasses. A piece of the semitendinous muscle was vacuum-packed and frozen (-20°C) until fatty acid analysis was performed.

### Fatty acid analysis

Fatty acids of intramuscular fat were extracted [56], methylated and analyzed with a gas chromatograph (Autosystem XL Agilent Technologies 7890 Net Work GC System, Perkin Elmer, Boston, USA) equipped with a flame ionization detector, a Hamilton injector, and an Omegawax 320 capillary column (30 m × 0.32 mm with a film thickness of 0.25 μm; Supelco, Bellefonte, USA) with He as the carrier gas at 30 cm/s. The temperature of the inlet detector was 260°C and the initial temperature of the oven was 190°C for 2 min, increasing to 205°C at a rate of 5°C/min for 3 min. Fatty acids were quantified using the internal standard (C21:0) after adjusting for the response as determined by Sigma-Aldrich standard mixtures (Sigma Aldrich, Madrid, Spain). Proportions of polyunsaturated (PUFA), monounsaturated (MUFA), and saturated (SFA) fatty acids, as well as n-6 and n-3 PUFA/SFA and n-6/n-3 ratios were obtained from individual fatty acid percentages.

### RNA extraction and cDNA synthesis

Total RNA was extracted from approximately 500 mg of semitendinous muscle using TRI REAGENT (Sigma Life Science) according to the manufacturer's instructions. The concentration and purity of the RNA were determined using nanophotometric analysis (Implen). To eliminate the possible amplification of contaminating genomic DNA, samples were treated with DNAse. Single-stranded cDNA was synthesized from 1 μg of RNA using the SuperScript III Reverse Transcriptase kit (Invitrogen), following the manufacturer's recommendations. Negative controls of cDNA synthesis reactions were conducted in the absence of reverse transcriptase and used as a template in PCR to verify the absence of genomic DNA contamination for each sample.

### Real-time polymerase chain reaction analysis (real-time RT-PCR)

Gene expression was analyzed by real-time RT-PCR (ABI Prism 7500 sequence detection system, Applied Biosystems, Madrid, Spain). According to the ovine *SCD*, *PPARγ *and *PPARα *cDNA sequences (GenBank Acc. Number AJ001048, AY137204, FJ200440 for *SCD*, *PPARγ *and *PPARα *respectively), primers for real-time RT-PCR were designed using the program Primer3 http://frodo.wi.mit.edu/primer3/. The sequences of the primers are shown in Table 3. The primers for *SCD *and *PPARγ *were designed across exon 4- exon 5 junction, while the primers for *PPARα *are designed in exon 7.

**Table 3:** Genes and real-time amplification products.

				PCR condition			
Target gene	Primers: Forward and reverse	Amplicon bp	Acc. Number	AT	Nm	R^2^	Slope
*SCD*	F-5'cccagctgtcagagaaaagg-3' R-5'gatgaagcacaacagcagga-3'	115	AJ001048	59	900 900	0.996	-3.35
*SREBP1*	F-5' ctgctatgcaggcagcac-3' R-5' ggttgatgggcagcttgt-3'	99	GU206528	59	900 900	0.980	-3.32
*PPAR*γ	F-5'cttgctgtggggatgtctc-3' R-5'ggtcagcagactctgggttc-3'	121	AY137204	60	900 900	0.982	-3.35
*PPARα*	F-5'tgccaagatctgaaaaagca-3' R-5' cctcttggccagagacttga-3'	99	FJ200440.1	59	300 300	0.98	-0.38
*ACTB*	F-5'ggacctgacggactacctcatg-3' R-5'ggccatctcctgctcgaagt-3'	136	U39357	60	300 300	0.989	-3.32
*SDHA*	F-5'catccactacatgacggagca-3' R-5'atcttgccatcttcagttctgcta-3'	90	AY970969	60	300 300	0.990	-3.36
*G6PDH*	F-5'tgacctatggcaaccgatacaa-3' R-5' ccgcaaaagacatccaggat-3'	76	DQ377364	60	900 900	0.994	-3.33
*UBC*	F-5 cgtcttaggggtggctgtta-3' R-5'aaattggggtaaatggctaga-3'	90	NM_001009202	59	600 600	0.991	-3.51

Genes, primers (F: forward and R: reverse), length of the amplicon and GenBank accession (acc.) numbers for the ovine sequences. Real-time RT-PCR conditions: annealing temperature (AT), primer concentrations (Nm), correlation coefficient (R^2^) and slope of the standard curve.

However, no ovine *SREBP1 *gene sequences have been deposited in GenBank. Primers designed from bovine genomic DNA (GenBank Acc. Number NC_007317.3) were used to obtain partial genomic DNA regions of ovine *SREBP1*. Genomic DNA was amplified in a final volume of 25 μl containing 5 pmol of each primer 5'-CACTTCATCAAGGCAGACTC-3' and 5'-GAGCTCAAGGAGACTGGTGGT-3', 200 nM dNTPs, 2 mM MgCl_2_, 50 mM KCl, 10 mM Tris-HCl, 0.1% Triton X-100 and 0.5 U Taq polymerase (Taq, Biotools). PCR amplification conditions included an initial denaturation step of 94°C for 3 minutes, then 30 cycles of 94°C for 30 seconds, 58°C for 30 seconds, and then 72°C for 30 seconds. PCR products were sequenced using an ABI Prism 3700 (Applied Biosystems) and standard protocols. Sheep-specific primers were designed for real-time RT-PCR (Table 3). The primers for *SREBP1 *real time RT- PCR are designed across exon 6- exon 7 junction. Homology searches were performed with BLAST (National Center for Biotechnology Information: http://www.ncbi.nlm.nih.gov/BLAST/), confirming the identity of the amplified fragment. Sheep-specific primers and the conditions for real-time RT-PCR are shown in Table 3.

Before performing the real-time RT-PCR reactions, a conventional PCR was carried out for *SCD*, *SREBP1, PPARα *and *PPARγ *genes with the aim to test the primers and verify the amplified products. PCR products were sequenced to confirm the gene identity using an ABI Prism 3700 (Applied Biosystems) and standard protocols.

The PCR was carried out in a total of 10 μl PCR mixture, containing SYBR Green PCR Master Mix (Applied Biosystems). Each reaction was run in triplicate and the average was used to calculate the relative amount of the target gene. Four housekeeping genes ovine beta actin (*ACTB*), succinate dehydrogenase (*SDHA*), glucose-6-phosphate dehydrogenase (*G6PDH*) and ubiquitin (*UBC) *stability were tested using geNorm software version 3.4., which calculates the measure of gene expression stability (M) of a putative reference gene based on the average pairwise variation between all investigated reference genes. The most stable genes were *ACTB *(M = 0,82) and *G6PDH *(M = 0,82) and the less stable *UBC *(M = 1,4). The geometric mean of the 3 most stable housekeeping genes were used to normalize each set of results *ACTB*, *G6PDH *and *SDHA *(M= 1,18) (4).

The relative gene expressions were normalized against a factor that was based on the geometric mean of the expression levels of the three housekeeping genes, according to the recommendation of Vandesompele *et al*. [57]. The amplification conditions were an initial step of 10 min at 95°C, followed by 40 cycles of 95°C for 15 s and 59°C for 30 s. The specificity of the amplification products was determined using a melting curve in all cases. The efficiency of the PCR amplification for each gene was calculated using the standard curve method (E= 10^-1/slope ^-1). The standard curves for each gene were generated by a five-fold serial dilution of pooled cDNA. Standart curve method was used to quantify the relative gene expression [58]. Normalized qPCR data were transformed in fold- change relative to group ALF. Normalized qPCR data were transformed to obtain a perfect mean of 1 in ALF group. PCR-normalized data are presented as *n*-fold change relative.

### Statistical analyses

Statistical analyses were carried out using the SPSS statistical software package, version 15.0. The normal distributions of all continuous variables were checked by the Kolmogorov-Smirnov test. To evaluate the effects of feeding system on fatty acid content, the analyses were carried out using a one-way ANOVA with post hoc Bonferroni correction for multiple comparisons.

The relative differences in *SCD, SREBP1, PPARα *and *PPARγ *gene expression between the different treatments were defined as the relative quantities after normalization. The relative differences between the groups were calculated and defined as the relative increase, setting the control means at 100%. A nonparametric U-Mann Whitney test was used to determine whether the differences observed between the effects of alimentation on *SCD, SREBP1, PPARα *and *PPARγ *gene expression were statistically significant (*P *< 0.05). Correlation analyses between the variables were performed using the Rho Spearman coefficient and were considered statistically significant when *P *< 0.05.

The association between the alimentation system and *SCD *gene expression with CLA content was carried out using the General Linear Model (GLM) in SPSS.15, where the alimentation system was included as the fixed factor and *SCD *expression as a covariate.

The equation of the model used was: y_ijk _= μ + A_j _+ b(E_k_) + (A*b(E))_jk _+ e_ijk_, where y_ijk _= CLA at observation; μ = overall mean; A_j _= effect of the feeding system (j = ALF, ALF+S, IND-GRE and IND); b(E_k_) = *SCD *gene expression, b the linear regression coefficient of y on gene expression; (A*b(E))_jk _= the effect of the interaction between the feeding system and *SCD *gene expression; and e_ijk _= the residual error. To investigate contributors of variation on *SCD *gene expression, analysis of variance was performed. Feedind system was included as fixed effect, and *SREBP1 *and *PPARγ *gene expression as covariates.

## Authors' contributions

ED has conducted research, and the statistical analysis. ED, MJ and JHC have written the manuscript. CS and CR have contributed in real time PCR and manuscript revision. MS has contributed in the statistical analysis and in manuscript revision. MJ and JHC designed research. MJ has provided animals. JHC has primary responsibility for final content. All authors have contributed in the manuscript discussion. All authors read and approved the final manuscript.
